# The complete mitochondrial genome of *Pontederia crassipes*: using HiFi reads to investigate genome recombination and gene transfer from chloroplast genome

**DOI:** 10.3389/fpls.2024.1407309

**Published:** 2024-06-28

**Authors:** Zhigang Hao, Xiaoqi Jiang, Lei Pan, Jingyuan Guo, Yi Chen, Jianqiang Li, Biao Liu, Anping Guo, Laixin Luo, Ruizong Jia

**Affiliations:** ^1^ Sanya Research Institution, Chinese Academy of Tropical Agriculture Sciences/Hainan Key Laboratory for Biosafety Monitoring and Molecular Breeding in Off-Season Reproduction Regions, Sanya, Hainan, China; ^2^ Hainan Seed Industry Laboratory, Sanya, Hainan, China; ^3^ Department of Plant Pathology, China Agricultural University, Beijing, China; ^4^ Sanya Institute of China Agricultural University, Sanya, China; ^5^ CAIQ Center for Biosafety in Sanya, Sanya, Hainan, China; ^6^ Ministry of Ecology and Environment, Nanjing Institute of Environmental Sciences, Nanjing, China

**Keywords:** *Pontederia crassipes*, Mitogenome, MTPT, RNA editing, phylogenetic analyses

## Abstract

Water hyacinth (*Pontederia crassipes* Mart.) is a monocotyledonous aquatic plant renowned for its rapid growth, extensive proliferation, biological invasiveness, and ecological resilience to variations in pH, nutrients, and temperature. The International Union for Conservation of Nature (IUCN) has listed *P. crassipes* among the top 100 invasive species. However, comprehensive genomic information, particularly concerning its mitochondrial genome (mitogenome), remains surprisingly limited. In this study, the complete mitogenome of *P. crassipes* was analyzed using bioinformatics approaches. The mitogenome is 399,263 bp long and contains 38 protein-coding genes (PCGs), 24 tRNA genes, and 3 rRNA genes. Sequence analysis revealed that the complete mitogenome of the species contains 3,289 dispersed repeats, and 765 RNA editing sites in protein-coding genes. The *P. crassipes* mitogenome possessed un-conserved structures, including extensive sequence transfer between its chloroplasts and mitochondria. Our study on the mitogenome of *P. crassipes* offers critical insights into its evolutionary patterns and phylogenetic relationships with related taxa. This research enhances our understanding of this invasive species, known for its significant biomass and rapid overgrowth in aquatic environments.

## Introduction

*Pontederia crassipes* (Mart.), known as the water hyacinth, is a monocotyledonous aquatic plant that floats on water. It belongs to the *Pontederia* genus within the Pontederiaceae family (Ben Bakrim et al., 2022). This species is native to the tropical and warm temperate regions of the Americas. It is known for its rapid growth, extensive proliferation, and remarkable resilience to fluctuations in pH, nutrient availability, and temperature. Many countries, including China, have introduced *P. crassipes* as a feed plant, medicinal plant, aquaponic plant, or for pollution control. Due to its aggressive nature, the International Union for Conservation of Nature has listed it among the top 100 invasive species, and it is also recognized as one of the world’s ten worst weeds (Ayanda et al., 2020). *P. crassipes* poses a threat not only to aquatic life but also to local communities. By blocking sunlight, it hampers the productivity of phytoplankton and other macrophytes, indirectly impacting the health of other aquatic organisms. This dominance results in diminished biodiversity in the habitats it invades, threatening various ecosystems in tropical and subtropical regions globally (Ayanda et al., 2020). Some research suggests potential benefits of *P. crassipes*, including its capacity to absorb heavy metals and thrive in polluted waters, positioning it as a viable phytoremediation agent for wastewater treatment. Traditionally, this plant has been used in remedies for gastrointestinal, including diarrhea, intestinal worms, digestive disorders, and flatulence. Furthermore, it has been explored as a promising source for both bioenergy and biofertilizers (Mishra and Maiti, 2017).

Mitochondria are complex organelles that play a central role in energy metabolism, control of stress responses, and serve as a hub for biosynthetic processes. They originated from symbiotic bacteria and have co-evolved with their host organisms (Roger et al., 2017). For most spermatophytes, nuclear genomic information is derived from both parents, while the mitogenome is predominantly maternally inherited. This genetic mechanism eliminates the influence of paternal-related information, simplifying genetic research. Mitochondria play a critical role in plant growth and development, being involved in fundamental cellular processes (Kroemer and Reed, 2000; van Loo et al., 2002; Bonora et al., 2014). Some research suggests a correlation between uncommon open reading frames (ORFs) in plant mitogenomes and cytoplasmic male sterility (CMS), a phenomenon that results in non-functional pollen (Chen and Liu, 2014). These CMS-associated ORFs have been identified in some plant species. Harnessing CMS in hybridization technology can potentially produce progeny with superior characteristics (Chen and Liu, 2014). One notable feature of plant mitogenomes is their elevated mutation rate, primarily attributed to the lack of efficient DNA repair systems. Furthermore, several plant mitogenomes have gained genes through horizontal gene transfer from external organisms. This phenomenon is particularly pronounced in higher plants, which have incorporated multiple plastid sequences from chloroplasts. This evolutionary trajectory has spanned extensive periods and is presumably an ongoing process (Choi and Park, 2021; Garcia et al., 2021; Lin et al., 2022). Deep sequencing of the mitogenome is a prerequisite for mitogenome editing and research on CMS. However, resources for plant mitogenomes are still very limited.

As of January 2024, over 13,000 plastomes from plants have been cataloged in the GenBank database. In contrast, only 673 mitogenomes of plants have been recorded, reflecting the challenges posed by the diversity of mitochondrial structures. The sizes of these mitogenomes range from 66 kb to 12 Mb (Wang et al., 2024). Most plant mitogenomes are circular, but some are linear. There are large numbers of sequence rearrangement in the mitogenome, which can lead to multiple configurations of the genome, for example, *Scutellaria tsinyunensis* with two conformations (Li et al., 2021). In addition, RNA editing events occur in the mitogenome, representing a post-translational modification phenomenon that results in differences between sequencing and template sequences (Fan et al., 2019; Yang et al., 2023; Jiang et al., 2023b).

In this study, we sequenced the *P. crassipes* mitogenome and characterized its structures and sequence features. To explore sequence migration between the chloroplast genome and mitogenome, we utilized the same data for chloroplast genome assembly. Additionally, RNA editing was analyzed and verified using lncRNA-seq data and PCR experiments. The obtained mitogenome serves as a valuable resource for future evolutionary analysis and functional research.

## Materials and methods

### Plant material and DNA sequencing

The fresh leaves of *P. crassipes* (Mart.) Solms were collected from Yazhou, Hainan, China. This species is native to the tropical and warm temperate regions of the Americas and is an invasive plant in China. We identified it according to the Flora of China (http://www.efloras.org/florataxon.aspx?flora_id=2&taxon_id=200027394, Vol. 24 Page 41) (Wu and Charles, 2000). A specimen has been deposited in the herbarium of the Chinese Academy of Tropical Agriculture Sciences in Hainan, China, with the accession number NFBIO-01-SY-0511. Total genomic DNA (gDNA) was extracted using the Tiangen Biotech DNA kit (Beijing, China), and then used to construct a DNA library with an insert size of 350 bp. A total of 20 Gb raw data was produced by DNA nanoball sequencing (DNBSQ). The constructed DNA library was produced on the DNBSEQ platform (MGI, China). We applied Trimmomatic (Bolger et al., 2014) to remove low-quality sequences, including those with a quality value (Q) of less than or equal to 5, which accounted for more than 50% of the total bases, as well as sequences containing more than 10% “N” bases. The gDNA was also subjected to sequencing on a PacBio Sequel II platform (Pacific Biosciences, USA), generating about 10Gb data.

### Organelle genome assembly

To obtain fully assembled plastome, we utilized GetOrganelle (version 1.7.4.1) with the following parameters: ‘-R 15 -k 21,45,65,85,105 -F embplant_pt’ to assemble the short-reads of gDNA (Jin et al., 2020). GetOrganelle generated two complete plastome sequences, and we selected the one that the SSC (small single-copy) region aligns in the same direction as *Arabidopsis thaliana* (NC_000932.1).

Subsequently, we performed *de novo* assembly of *P. crassipes* long-reads using Flye (v.2.9.1-b1780) (Kolmogorov et al., 2019) with the parameters ‘–min-overlap 2,000’. The graphical fragment assembly in GFA format are obtained. For all the obtained contigs, BLASTn program (Chen et al., 2015) was used to identify mitochondrial contigs based on the conserved plant mitochondrial genes in *A. thaliana* (NC_037304.1). The parameter is “-evalue 1*e*-5 -outfmt 6 -max_hsps 10 -word_size 7”. The GFA file was visualized by Bandage software (Wick et al., 2015).

### Verification of the mitogenome structure

The mitogenome structure of *P. crassipes* was investigated using PCR experiments with specific primers (**Supplementary Table S1**) to verify the accuracy of assembly. Primer design was conducted using the Primer designing tool on NCBI (https://www.ncbi.nlm.nih.gov/tools/primer-blast/) with default parameters. The PCR reaction volume was 25 µL, containing 2 µL of template DNA, 0.5 µL of forward primer, 0.5 µL of reverse primer, and 12.5 µL of 2x Taq PCR Master Mix. The amplification procedure consisted of an initial denaturation at 94°C for 5 min, followed by 30 cycles of denaturation at 94°C for 30 s, annealing at 58°C for 30 s, extension at 72°C for 60 s, and a final extension step at 72°C for 5 min. PCR amplicons were visualized using 1% agarose gel electrophoresis. Subsequently, the PCR products were sequenced to verify the mitogenome structure.

### Annotation of organellar genomes

The plastome of *P. crassipes* was annotated using CPGAVAS2 (Shi et al., 2019) with another plastomes of the same species (NC_046773.1) serving as reference genomes. The annotation results were further verified using CPGView (Liu et al., 2023) to ensure accurate gene annotations. Then, we used IPMGA (http://www.1kmpg.cn/ipmga/) to annotate the assembled mitogenome of *P. crassipes*. The tRNA annotations were performed using tRNAscan-SE (Lowe and Eddy, 1997) while rRNA annotations were obtained through BLASTn (Chen et al., 2015). To ensure accuracy, manual edits were made to the annotations using Apollo (Lewis et al., 2002). Finally, the genome map was generated using OGDRAW (v1.3.1) (Alverson et al., 2010).

### Analysis of codon usage

We employed PhyloSuite (v1.2.2) (Zhang et al., 2020) to parse the GenBank format file of the *P. crassipes* mitogenome, and extracting the protein-coding genes (PCGs). Subsequently, we conducted an analysis of the codon usage in mitochondrial PCGs using Mega 7.0 (Kumar et al., 2016), which involved the calculation of Relative Synonymous Codon Usage (RSCU) values.

### Repeat element analysis

The simple sequence repeats (SSRs) were identified using the online tool MISA (https://webblast.ipk-gatersleben.de/misa/). The parameters for the minimum numbers of mono-, di-, tri-, tetra-, penta-, and hexanucleotides were set as 10, 5, 4, 3, 3, and 3, respectively. Long tandem repeats were detected using Tandem Repeats Finder (TRF) with the default parameters (https://tandem.bu.edu/trf/trf.html). Additionally, forward, reverse, and palindromic repeat sequences were identified using REPuter (Benson, 1999) with the following settings: hamming distance of three and minimal repeat size of 30 bp, and e-value is limited to less than 1*e*-05. The visualization of the repetitive elements was done using the Circos (Zhang et al., 2013).

### Identification of the mitochondrial plastid sequences (MTPTs)

To identify the MTPTs, homologous fragments between the plastome and mitogenome of *P. crassipes* were analyzed using BLASTn software. The analysis was conducted with the following parameters: -evalue 1e-5, -word_size 9, -gapopen 5, -gapextend 2, -reward 2, -penalty -3. The results were visualized using Circos (Zhang et al., 2013). For the two MTPTs located on the inverted repeat regions of the plastome, we count only one time.

### Collinear analysis

For the collinear analysis involving *P. crassipes*, we selected five related species: *Carex breviculmis* (NC_068626.1), *Cyperus esculentus* (NC_058697.1), *Phoenix dactylifera* (MH176158.1), *Phoenix dactylifera* (NC_016740.1), and *Cocos nucifera* (NC_031696.1). Collinear blocks were identified based on sequence similarity using the BLASTn program with the following parameters: -evalue 1*e*-5, -word_size 9, -gapopen 5, -gapextend 2, -reward 2, -penalty -3. Only collinear blocks longer than 1 kb were retained for downstream analysis. To visualize the collinear relationships, we generated a multiple synteny plot using TBtools (Chen et al., 2023).

### Phylogenetic analysis

We retrieved a total of 29 mitogenomes from the GenBank database (https://www.ncbi.nlm.nih.gov/), including two outgroups (*Asparagus officinalis* and *Chlorophytum comosum*). Firstly, PhyloSuite (v.1.2.2) (Zhang et al., 2020) was used to identify and extract orthologous protein-coding genes (PCGs) across the analyzed species. The nucleotide sequences corresponding to these PCGs were then aligned using MAFFT (v7.471) (Katoh and Standley, 2013). Subsequently, the aligned sequences were concatenated to generate the input for phylogenetic tree construction. The maximum likelihood (ML) method was implemented using IQ-TREE (version 2.1.4-beta) (Minh et al., 2020) with the parameters “–alrt 1000 -B 1000”. Bootstrap analysis was performed with 1,000 replicates. Finally, the resulting phylogenetic tree was visualized and edited using the online tool iTOL (Letunic and Bork, 2019).

### RNA extracting and sequencing

To characterize RNA editing sites occurring on mitochondrial transcripts, we employed a library construction strategy specific for long non-coding RNAs (lncRNAs). This approach avoids bias towards sequencing transcripts rich in polyA tails, thereby significantly enhancing the abundance of mitochondrial transcripts. To extract lncRNAs, total RNA was obtained from freshly harvested *P. crassipes* leaves using a high-quality RNA extraction kit (TRIzol^®^ Reagent, Thermo Fisher Scientific, USA) following the manufacturer’s guidelines. For prokaryotic or lnc library, mRNA was purified from total RNA using probes to remove rRNA. Fragmentation was carried out using divalent cations under elevated temperature in First Strand Synthesis Reaction Buffer (5X). First strand cDNA was synthesized using random hexamer primer and M-MuLV Reverse Transcriptase (RNase H). Second strand cDNA synthesis was subsequently performed using DNA Polymerase I and RNase H. Remaining overhangs were converted into blunt ends via exonuclease/polymerase activities. After adenylation of 3’ ends of DNA fragments, NEBNext Adaptor with hairpin loop structure were ligated to prepare for hybridization. In order to select cDNA fragments of preferentially 370~420 bp in length, the library fragments were purified with AMPure XP system (Beckman Coulter, Beverly, USA). The integrity and concentration of RNA were evaluated using the Agilent 2100 Bioanalyzer (CA, USA) and NanoDrop spectrophotometer (MA, USA). The enriched lncRNA was subsequently used in the construction of a cDNA library following a protocol tailored for lncRNA sequencing. The resulting library was sequenced on an Illumina NovaSeq 6000 platform (Illumina, USA). Quality control measures were implemented to remove sequences of low quality, and bioinformatics analyses were conducted on the resultant data to identify and characterize lncRNAs.

### Analysis of RNA editing sites

We predicted RNA editing sites by mapping transcriptome data (RNA-Seq) and genome data (WGS-Seq). Initially, we used BWA software (Li and Durbin, 2009) with default parameters to map RNA-Seq reads to the mitochondrial coding sequences (CDS) of each protein-coding gene (PCG). Subsequently, we employed REDItools (Picardi and Pesole, 2013) to identify RNA editing sites based on the BWA mapping results. The prediction thresholds were set as follows: coverage greater than 30 bp, frequency more than or equal to 0.1, and p-value less than or equal to 0.05. Next, we mapped DNA-seq reads to the CDS of each PCG using BWA software with default parameters. Duplicate mappings were removed using SAMtools (version 1.6). We used the BCFtools (Li, 2011) to identify single nucleotide polymorphisms (SNPs) based on the DNA-seq mapping results, with the following thresholds: coverage greater than 30 and frequency less than or equal to 0.1. The purpose of identifying SNPs was to exclude polymorphisms in the genome sequence itself (focusing on C and T polymorphisms), as these sites should not be considered RNA editing sites. Finally, we extracted sites that were not identified as SNPs, representing the RNA editing sites in the protein-coding genes of *P. crassipes*.

### The validation of RNA editing sites

We observed that some RNA editing sites generated new start codons (*nad1* and *rps10*) and new stop codons (*atp6* and *atp9*). To confirm the accuracy of the identified RNA editing sites, we designed experiments to validate these four specific sites. Primers (**Supplementary Table S1**) were designed on both sides of the editing sites, and amplification was performed using genomic DNAs (gDNAs) and complementary DNAs (cDNAs) as templates. The reaction conditions for amplification are described above. The amplified products were subsequently subjected to Sanger sequencing. Ultimately, a comparison of sequences derived from gDNAs and cDNAs was performed to ascertain the presence of RNA editing events.

## Results

### Genomic structure of the *P. crassipes* mitogenome

**Figure 1:**
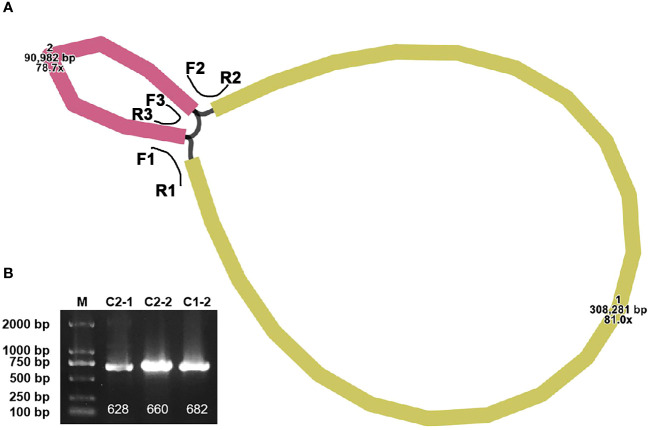
The assembly graph of the *P. crassipes* mitogenome and validation. **(A)** The *P. crassipes* mitogenome consists of two contigs (contig1 with 308,281 bp and highlighted in brown color, and contig2 with 90,982 bp and highlighted in yellow color, respectively). The three primers, F1-R1, F2-R2, and R3-R3, represented three protentional paths. **(B)** PCR validation of the three protentional paths, the sanger sequencing results fully illustrated in **Supplementary Figure S2**.

The completed mitogenome measures 399,263 bp and comprises two contigs (**Figure 1A**). An overlapping region between the two contigs is evident along their connecting lines. Specifically, contig1 measures 308,281 bp, while contig2 is 90,982 bp. The representation in **Supplementary Figure S1** suggests that there are two configurations in the mitogenome of *P. crassipes*. Configuration 1 indicates that contig1 can form a circular structure with contig2. Additionally, configuration 2 suggests that contig2 is capable of independently forming its own circular structure, while contig1 assumes a linear configuration. To confirm the presence of these two configurations within the *P. crassipes* mitogenome, we conducted PCR experiments. In this experiment, we designed three pairs of primers, as listed in **Supplementary Table S1**. Two of these pairs (F1 + R1, F2 + R2) were used to confirm that contig1 and contig2 can form a circular structure together. The third pair (F3 + R3) was employed to validate that contig2 can form an independent circular structure. The PCR amplification results showed bands with lengths consistent with those expected (**Figure 1B**), and Sanger sequencing results (**Supplementary Figure S2**) confirmed these configurations are correct. In the subsequent analyses, we adopted configuration 1 as the master circles of the *P. crassipes* mitogenome. This decision was made because only configuration 1 represents a classic circular DNA structure, where all sequences can be displayed on a single DNA molecule. Therefore, we opted for this configuration.

### General feature of the *P. crassipes* mitogenome

The mitogenome of *P. crassipes* comprises a total of 38 protein-coding genes (PCGs) (**Table 1**), which include 6 ATP synthase genes, 4 cytochrome c biogenesis genes, 9 NADH dehydrogenase genes, 3 cytochrome *c* oxidase genes, 1 transport membrane protein gene, 1 maturase gene, 1 cytochrome *b* gene, and 1 succinate dehydrogenase gene. Additionally, it contains 3 large subunits of ribosomal proteins and 9 small subunits of ribosomal proteins. A total of 24 tRNA genes have been annotated; however, some tRNA genes have multiple copies. After removing these duplicates, there are only 18 different tRNA genes. Among these, 9 tRNA genes are native to the mitochondria. Furthermore, our investigation has revealed 7 tRNA genes originating from the plastid: two copies of *trnN-GUU*, *trnH-GUG*, *trnR-ACG*, *trnA-UGC*, *trnL-CAA*, and *trnW-CCA*. Notably, our exploration has led us to the identification of two tRNA genes with bacterial origins, that is the two copies of *trnC-GCA*, exhibiting a remarkable level of sequence homology with previously documented genes (Kitazaki et al., 2011). The remaining tRNA genes, which lack sequence homology with known organelle tRNA genes, are of unknown origin (Rice et al., 2013). Additionally, we have successfully identified 3 rRNA genes within the *P. crassipes* mitogenome, namely *rrn5*, *rrn18*, and *rrn26*.

**Table 1:** Gene composition in the mitogenome of *P. crassipes*.

Group of genes	Name of genes
ATP synthase	*atp*1, *atp*4, *atp*6, *atp*8, *atp*9
NADH dehydrogenase	*nad*1, *nad*2, *nad*3, *nad*4, *nad*4L, *nad*5, *nad*6, *nad*7, *nad*9
Cytochrome *b*	*cob*
Cytochrome *c* biogenesis	*ccmB, ccmC, ccmFC, ccmFN*
Cytochrome *c* oxidase	*cox*1, *cox*2, *cox*3
Maturases	*mat*R
Transport membrane protein	*mtt*B
Succinate dehydrogenase	*sdh*3
Ribosomal protein large subunit	*rpl2, rpl*5, *rpl*16
Ribosomal protein small subunit	*rps1, rps2, rps3, rps4, rps7, rps10, rps12, rps13, rps14*
Ribosome RNA	*rrn5*, *rrn18*, *rrn26*
Transfer RNA	*trnQ-UUG, trnM-CAU, trnS-GCU, trnR-ACG, trnN-GUU, trnC-GCA, trnK-UUU, trnN-GUU_copy2, trnD-GUC, trnI-CAU, trnE-UUC, trnC-GCA_copy2, trnfM-CAU, trnP-UGG, trnF-GAA, trnF-GAA_copy2, trnS-GCU_copy2, trnH-GUG, trnL-CAA, trnW-CCA, trnL-CAA_copy2, trnQ-UUG_copy2, trnS-UGA, trnY-GUA*

**Figure 2:**
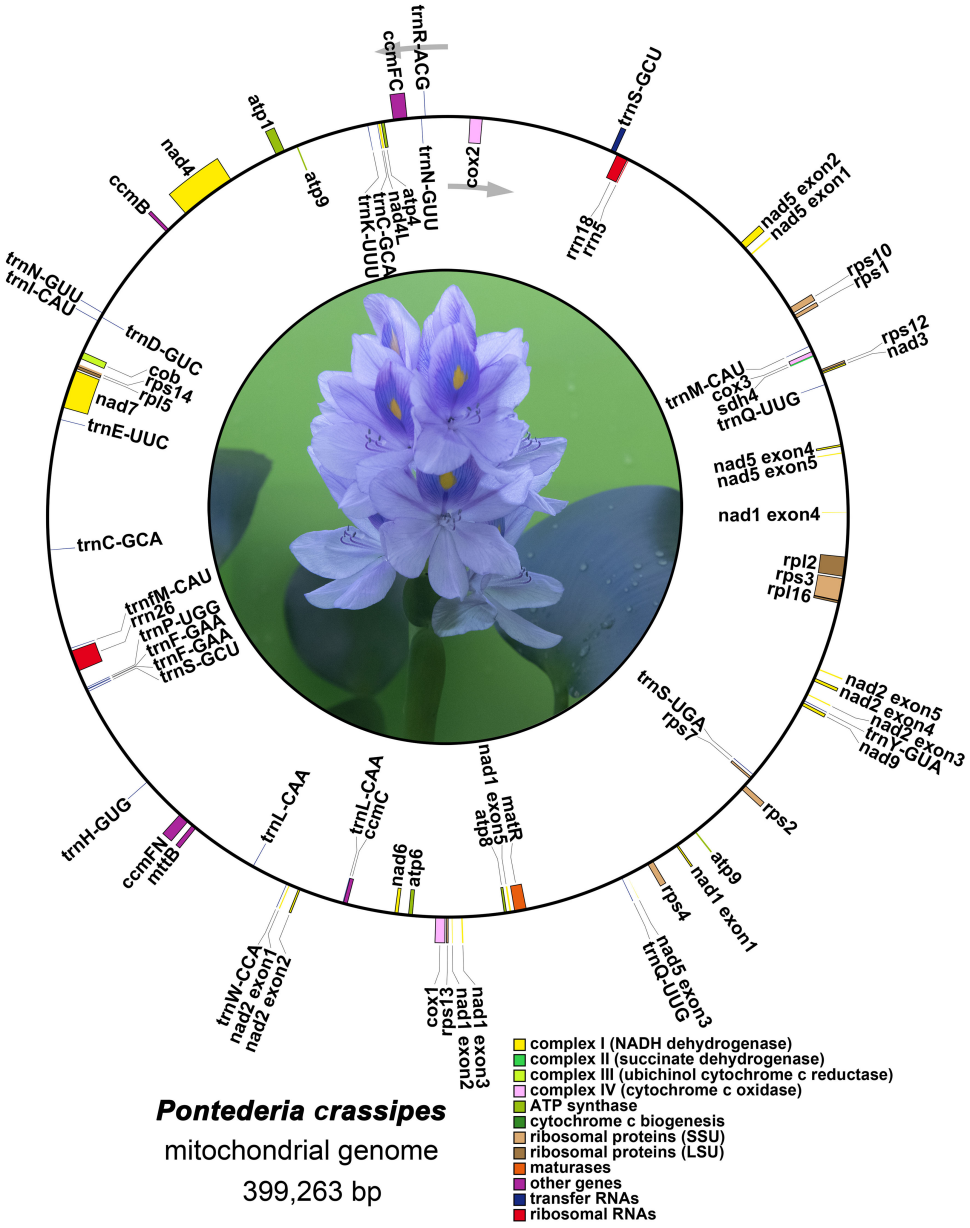
The mitogenome map of *P. crassipes*. Genes transcript clockwise marked outside the circle, and genes transcript counter-clockwise marked inside the circles, respectively. Genes belonging to different functional category are color-coded.

The precise positions of each gene are depicted in the mitogenome maps of *P. crassipes* (**Figure 2**; **Supplementary Table S2**). Among the protein-coding genes (PCGs), the genes *ccmFC*, *cox2*, *rps3*, *rpl2*, *trnS-GCU*, and *rps10* possess 1 intron each, while *nad4* possesses 3 introns, and the genes *nad1*, *nad2*, *nad5*, and *nad7* possess 4 introns each.

Additionally, we compared our newly sequenced mitogenome (OR680719.1) with another mitogenome of the same species released in GenBank (PP112345.1). In terms of gene annotation, the number of annotated genes in both mitogenomes is identical, demonstrating conservation of gene numbers. Since the orientation and starting points of the two sequences were inconsistent, we manually reverse-complemented PP112345.1 and set position 279,646 of the reverse-complemented sequence as the new starting point to ensure alignment of the two mitogenome sequences. We aligned the sequences using the default parameters of MAFFT (Katoh and Standley. 2013) and found that the two sequences exhibit homology in genomic structure, with no genomic rearrangements detected. The aligned sequence length is 402,198, and it can be found in **Supplementary File 1**. The dot-plot drawn by the online version of MAFFT (https://mafft.cbrc.jp/alignment/server/) is provided in **Supplementary Figure S3**. Using DnaSP (version 6.0) (Rozas et al., 2017) for analysis, we identified 75 indels and 325 single nucleotide polymorphisms (SNPs). These SNPs and indels are all located in non-coding regions. Among them, two regions show the highest frequency of variations: one from 261,665 to 269,827, containing 71 SNPs and 27 indels, and the other from 295,812 to 299,004, containing 170 SNPs and 21 indels (**Supplementary Table S3**). These results indicate a high degree of conservation among individuals of *P. crassipes* mitogenomes, with two hotspot regions where variations are concentrated.

### Simple sequence repeats analysis

**Figure 3:**
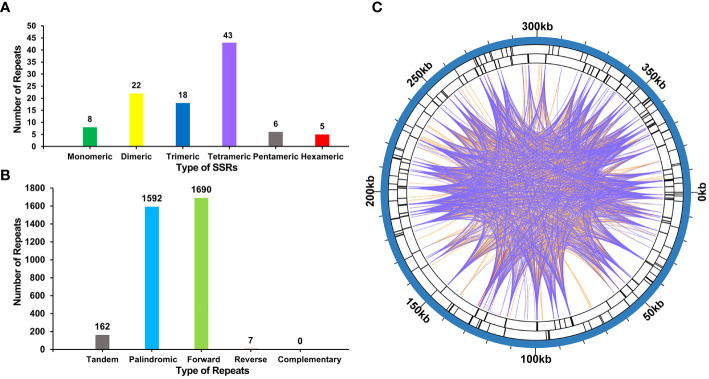
The number of SSRs, tandem repeat sequence and dispersed repeat sequences in *P. crassipes* mitogenome. **(A)** The types of SSRs and frequency in *P. crassipes* mitogenome. **(B)** Types and frequency of dispersed repeat sequences and tandem sequences in *P. crassipes* mitogenome. **(C)** The distribution of identified dispersed repeat sequences in the mitogenomes of *P. crassipes*. The purple ribbons represent the forward repeats, the orange ribbons represent the palindromic repeats and the red ribbons represent the reverse repeats.

The mitogenome of *P. crassipes* contains a total of 102 simple sequence repeats (SSRs) (**Figure 3A**; **Supplementary Table S4**). Among these SSRs, tetrameric repeats are the most abundant (43), followed by dimeric repeats (22), trimeric repeats (18), monomeric repeats (8), pentameric repeats (6), and hexametric repeats (5). Additionally, 162 long tandem repeat elements were identified in the mitogenome of *P. crassipes* (**Figure 3B**; **Supplementary Table S5**). Furthermore, a total of 3,289 dispersed repeats were found within the *P. crassipes* mitogenome (**Figure 3B**; **Supplementary Table S6**). Most of these repeat elements are less than 300 bp in length, with the longest being 7,699 bp. Interestingly, the number of dispersed repeats exceeds that of both SSRs and tandem repeats. The total length of these dispersed repeats spans 227,902 bp, constituting 57.08% of the entire *P. crassipes* mitogenome (**Figure 3C**). This abundant presence of repeats suggests their potential significance in genome reconfiguration and influencing genome size dynamics.

### Codon usage analysis

We conducted an analysis of codon usage within the protein-coding genes (PCGs), where the relative synonymous codon usage (RSCU) value greater than 1 indicates a higher frequency of codon usage, while a value of 1 signifies a neutral preference for codon utilization. This trend is visually depicted in **Supplementary Figure S4**. The comprehensive analysis of codon usage in *P. crassipes* reveals a discernible preference for specific codons among mitochondrial PCGs (**Supplementary Figure S4**, **Supplementary Table S7**). Notably, the RSCU values for the start codons AUG (Met) and UGG (Trp) both equate to 1. The RSCU values for the termination codons UGA (End), UAA (End), and UAG (End) are recorded as 0.79, 1.32, and 0.88, respectively. Regarding specific codons, GCU (Ala), CAA (Gln), and CAU (His) emerge as the three most frequently employed codons within *P. crassipes*. Conversely, GCG (Ala), GGC (Gly), and UAC (Tyr) are identified as the three least utilized codons. Furthermore, the prevalence of arginine (Arg), leucine (Leu), and serine (Ser) codons is notable, while methionine (Met) and tryptophan (Trp) codons exhibit relatively lower occurrence rates.

### Identification of MTPTs

**Figure 4:**
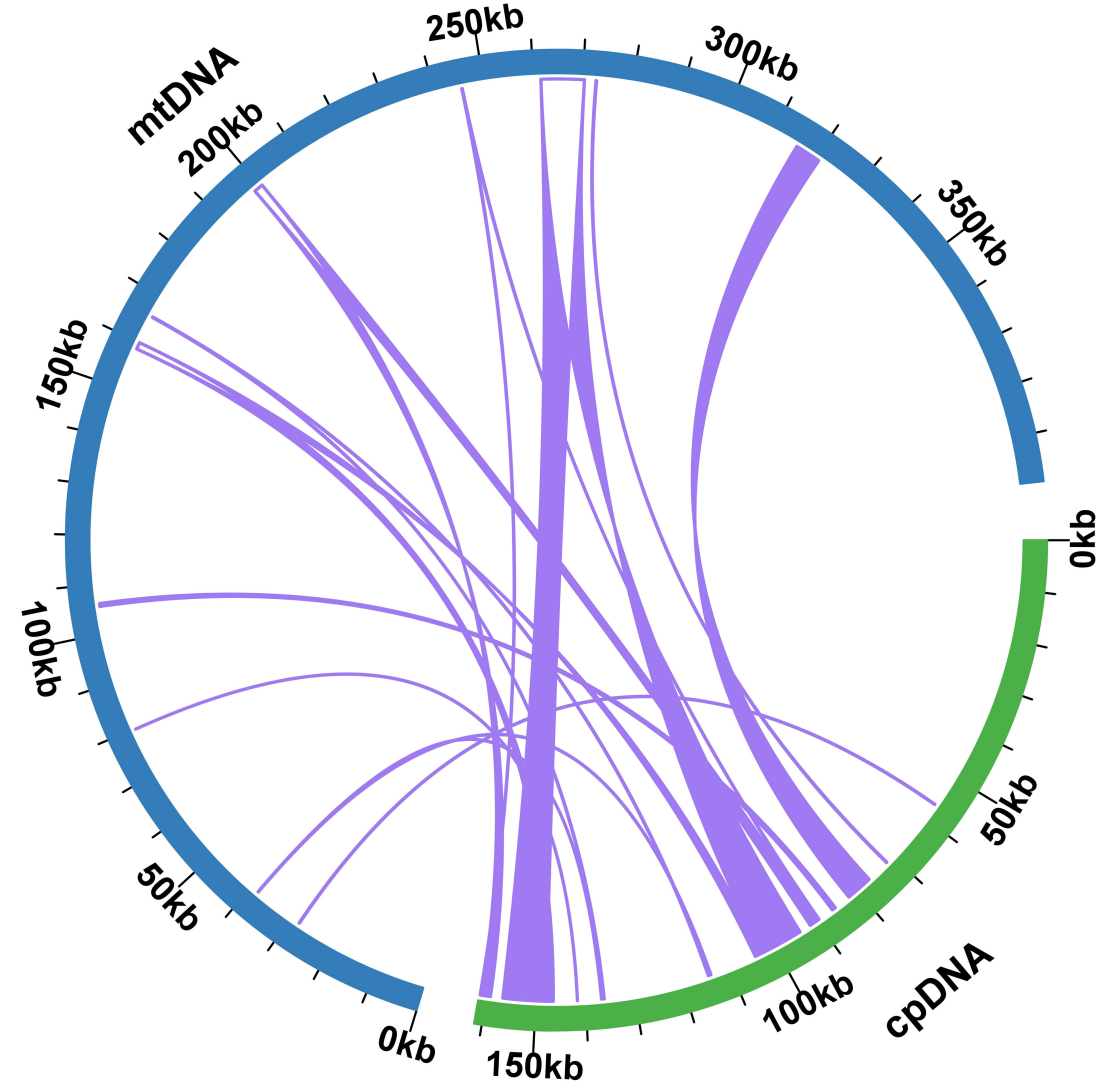
Schematic representation of the distribution of MTPTs between the mitogenome and the plastome of *P. crassipes*. The plastome indicated with green bar, and mitogenome indicated with blue bar. The MTPTs on the chloroplast IR regions were counted only once. The location of each MTPT has been marked accordingly.

Upon comparison between the plastidial and mitogenomes of *P. crassipes*, we identified 12 mitochondrial plastid DNA transfers (MTPTs) (**Figure 4**; **Supplementary Table S8**). These MTPTs collectively span a total length of 30,614 bp, constituting 7.68% of the entire mitogenome. The size of MTPTs ranges from 31 bp (MTPT3) to 8,767 bp (MTPT5). Further annotation of these MTPTs revealed the presence of plastidial genes or gene fragments within each MTPT. Notably, MTPT9 encompasses a series of plastid genes associated with the photosystem II protein complex, including *psbB*, *psbT*, *psbN*, and *psbH*. Additionally, sequence analysis unveiled that the migrated protein-coding genes (PCGs) experienced some degree of sequence loss, with only partial sequences detectable. This observation suggests potential non-functionality of these PCGs.

### RNA editing events and PCR validation

**Figure 5:**
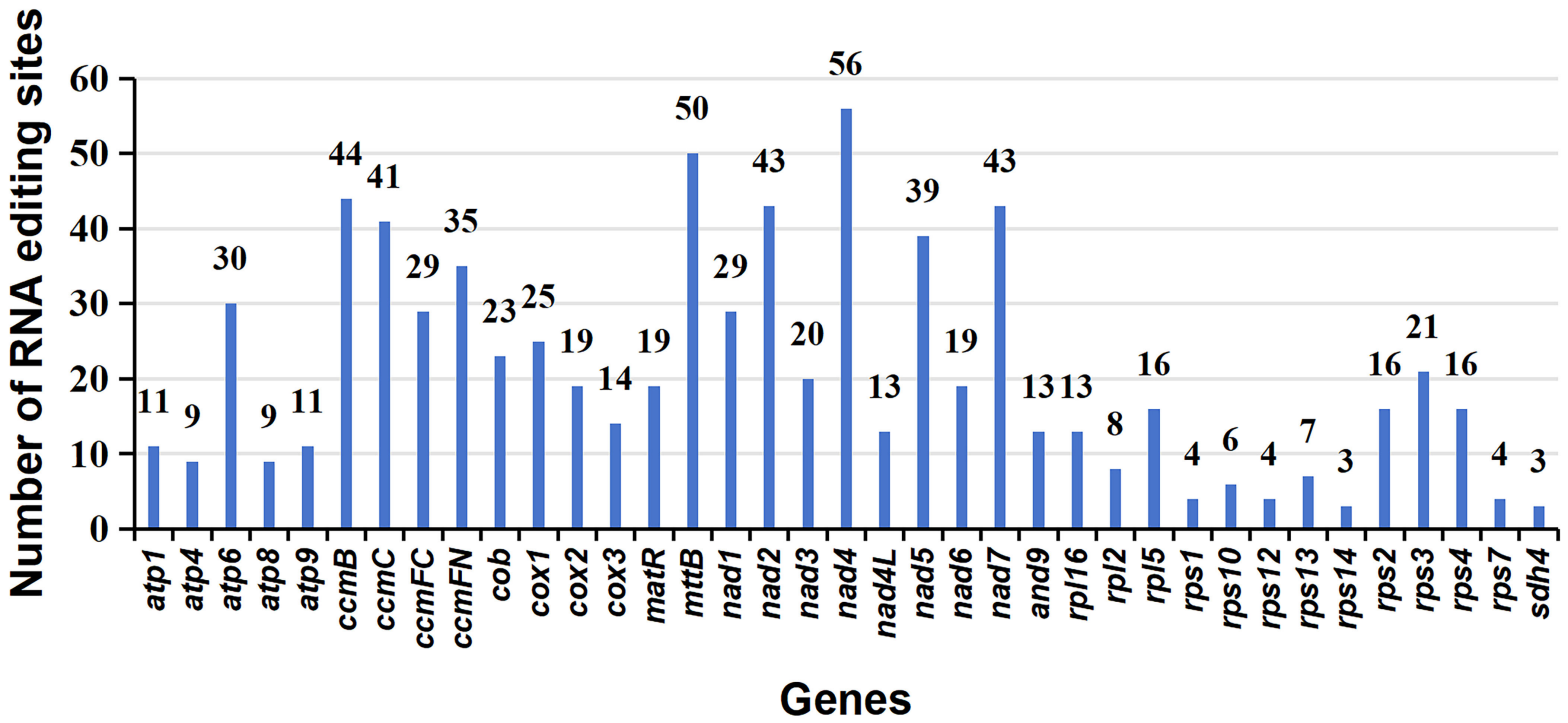
Characteristics of the RNA editing sites identified in mitochondrial PCGs of *P. crassipes.* The ordinate shows the number of RNA editing sites identified in PCGs, the abscissa shows the name of PCGs identified in the mitogenome of *P. crassipes*.

A total of 765 RNA editing sites (C to U) were identified within the mitogenome PCGs (**Figure 5**; **Supplementary Table S9**). Notably, *nad4* leads with the highest count of RNA editing sites (56), followed by *mttB* with 50 RNA editing sites, indicating these genes as primary targets in terms of RNA editing frequency. Our investigation highlights C to U RNA editing events in two genes, *atp6* (105/947, 0.11) and *atp9* (6145/6351, 0.97), leading to the introduction of premature stop codons. Interestingly, RNA editing also facilitates the formation of start codons, as observed in genes *nad1* (1482/2470, 0.6) and *rps10* (839/892, 0.94). In the provided data, the numbers in brackets represent the reads supporting RNA editing events, including the total number of reads at that site and the frequency of editing (**Supplementary Table S9**).

Four RNA editing genes (*nad1–2*, *rps10–2*, *atp6–718*, and *atp9–223*) were selected for validation using the PCR method (**Supplementary Figure S5**). Notably, the *rps10* gene, with an intron length of 758 bp, resulted in inconsistent lengths of amplified DNA fragments between gDNA and cDNA (**Supplementary Figure S5A**). Detailed primer sequences for these experiments can be found in **Supplementary Table S10**. Among these sites, *rps10–2* exhibited a clear RNA editing event, while the remaining three sites displayed distinct hybrid peaks of base C and U in cDNA compared to gDNA (**Supplementary Figure S5B**).

### Collinear analysis

**Figure 6:**
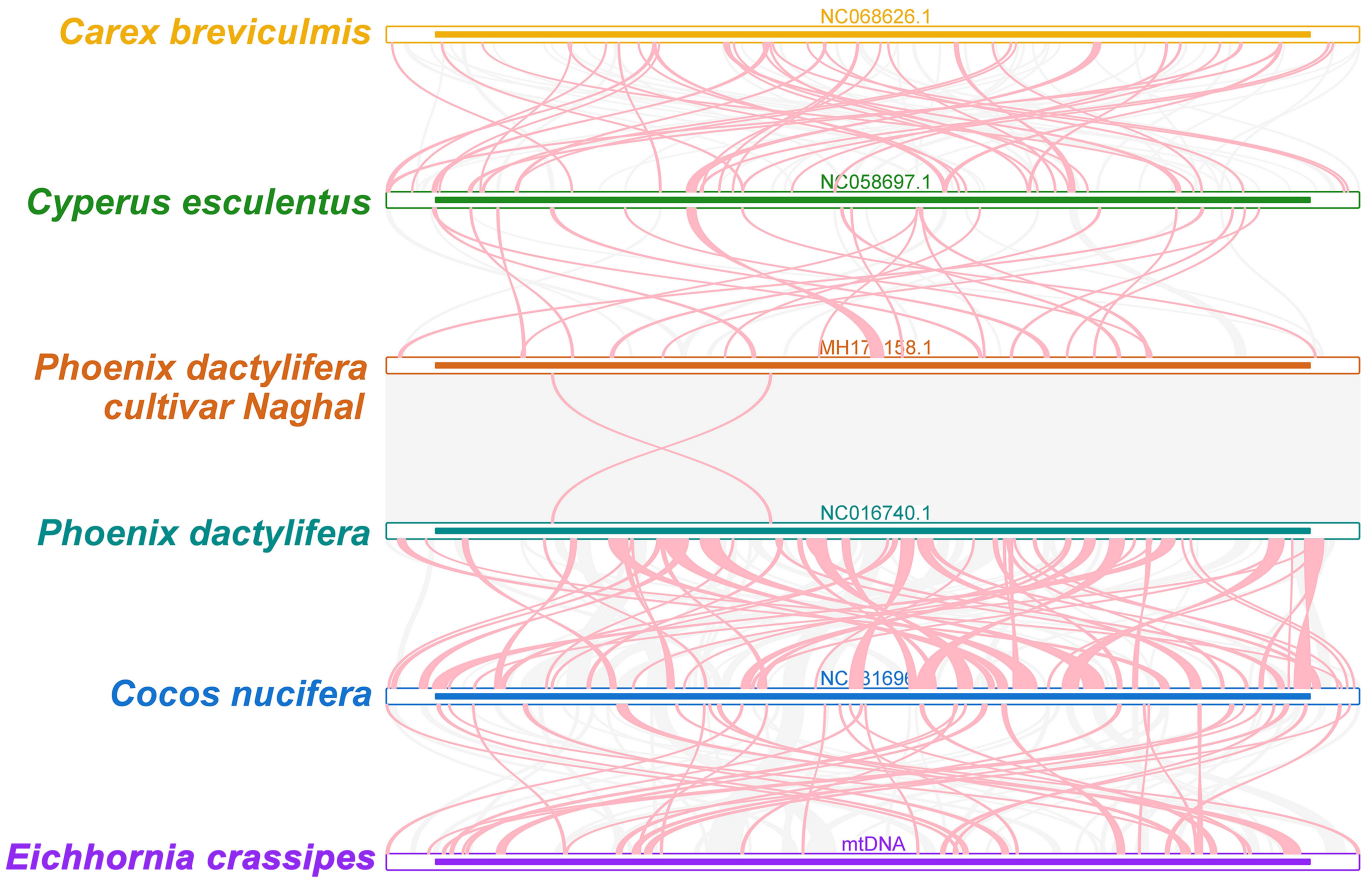
Collinear analysis of *P. crassipes* mitogenome and its related species. The colorful bars indicated the mitogenomes, and the ribbons showed the homologous sequences between the adjacent species. The homologous blocks less than 0.5 kb in length are not remaining, and regions that fail to have a homologous block indicate that they are unique to the species.

To explore rearrangements and conserved sequences within the *P. crassipes* mitogenome, we identified homologous collinear blocks (**Figure 6**). Comparative analysis between *P. crassipes* and *Cocos nucifera* revealed a lack of large adjacent collinear blocks and no collinear blocks surpassing 10 kb in length. Overall, the mitogenomes exhibited sparse collinearity with several non-homologous regions, indicating widespread genomic rearrangements between *P. crassipes* and related mitogenomes. Unique sequences were observed between individuals of different genera, while closely related species, such as the two cultivars of *Phoenix*, exhibited extremely high collinearity with minimal genome rearrangement detected.

### Phylogenetic analysis

**Figure 7:**
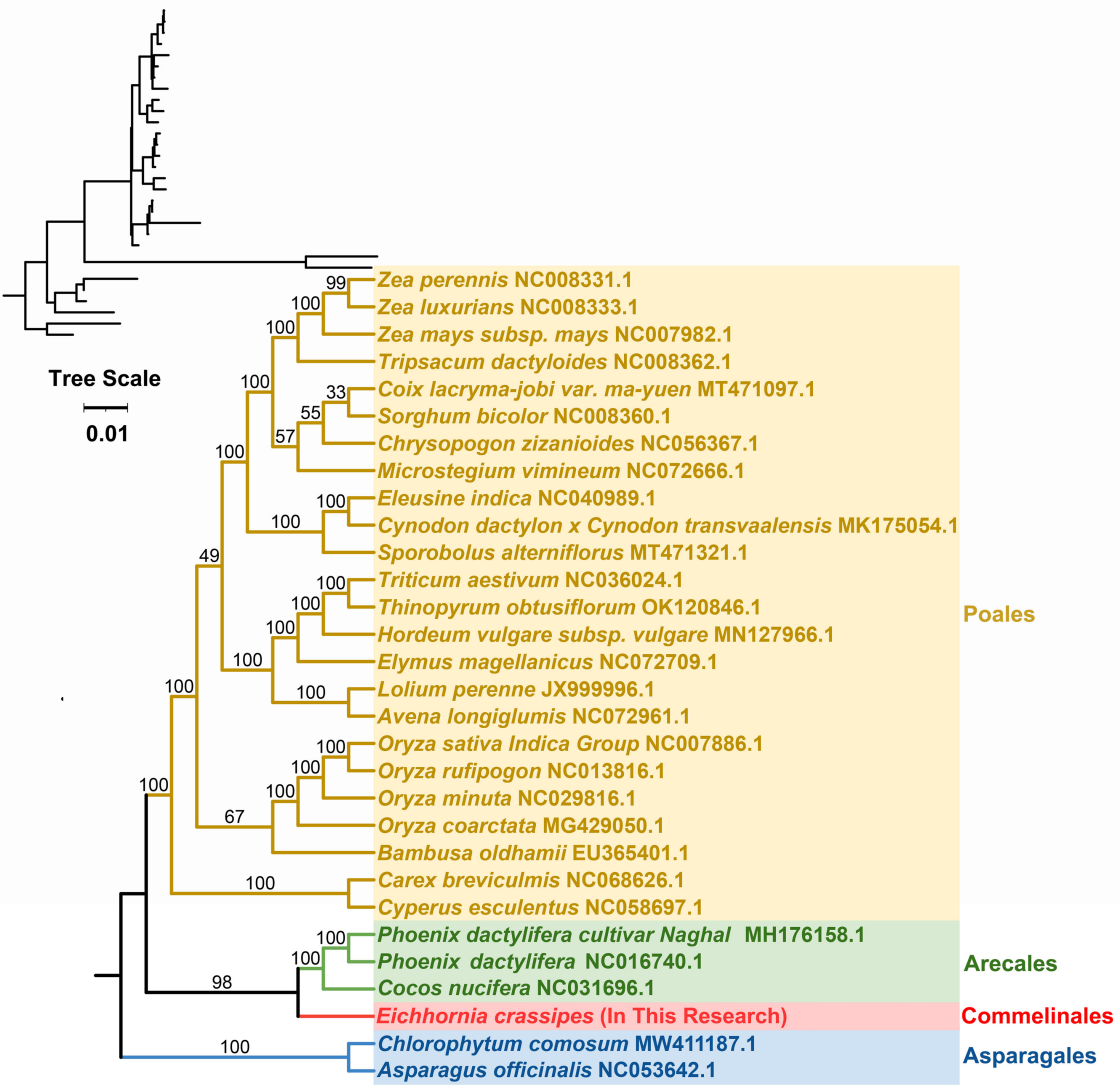
The phylogenetic relationships of *P. crassipes* and another 29 species based on conserved mitochondrial genes. The tree was constructed based on the nucleotide sequences of conserved mitochondrial protein-coding genes (PCGs). We used Maximum Likelihood (ML) method to reconstruct the phylogenetic tree. In the top left corner, we placed the original tree with branch length information, while the tree in the bottom right corner is the tree after processing, with branch lengths ignored. The ML topology is indicated with ML bootstrap support values. *C. comosum* and *A. officinalis* were used as outgroups.

A phylogenetic analysis with the mitogenomes of *P. crassipes* and 30 other angiosperm species were performed (**Figure 7**; **Supplementary Table S11**). *Asparagus officinalis* and *Chlorophytum comosum* serving as outgroups. The resulting Maximum Likelihood (ML) tree, depicted in **Figure 7**, demonstrates robust support for its primary basal branches, consistent with the latest classification by the Angiosperm Phylogeny Group (APG IV system). However, some lineages on the tree did not receive high support value, which may have been caused by potential horizontal gene transfer events in plant mitochondria, or the high conservation of the gene sequence. Previous studies suggest that specific plant mitochondrial genes may be inherited from other plants, resulting in tree topologies that deviate from true phylogenetic patterns (Garcia et al., 2021; Yu et al., 2021).

## Discussion

In contrast to the relatively conserved chloroplasts in plants, plant mitogenomes have undergone significant changes in their genomic structures. These mitogenomes exhibit intricate structures, encompassing polycyclic contigs, linear branches, and more. Assembling these complex mitogenomes correctly presents a formidable challenge (Chevigny et al., 2020). Such complexities might stem from recombination mediated by repeated sequences (Wang et al., 2023a). Numerous studies have delved into the intricacies of plant mitogenome structural variations, and several tools have been devised to decode these ever-evolving genomes (He et al., 2023; Shan et al., 2023). Our research focuses on the mitogenome of *P. crassipes*, which spans 399,263 bp. Notably, the *P. crassipes* mitogenome showcases a convoluted circular structure (**Figure 1**). When analyzing its dispersed repeat sequences, we observed that the vast majority (91.63%) are under 100 bp, with only one dispersed repeat sequence being 7,699 bp. Extensive research indicates that such short-dispersed repeats play important roles in mitogenome recombination across some plant species, including *Ginkgo biloba* (Guo et al., 2016), *Silene latifolia* (Sloan et al., 2012), and *Scutellaria tsinyunensis* (Li et al., 2021). In our study, the complex circular structure of the *P. crassipes* mitogenome appears to be influenced by this profusion of short dispersed repeat sequences. One study noted that the structure of the mitogenome can change dynamically within an individual. For instance, the *Vigna radiata* mitogenome exhibits a rosette shape under normal conditions but transitions to a linear form when exposed to cold temperatures (Lin et al., 2016). We conducted PCR experiments (**Figure 1B**) and Sanger sequencing (**Supplementary Figure S2**) to validate the structure of the *P. crassipes* mitogenome. The results unequivocally confirmed the presence of all linkages in the *P. crassipes* mitogenome assembly.

The genome structure and evolutionary processes of plant mitogenomes predispose them to readily absorb and incorporate foreign DNA. While plastid DNA is relatively conservative, plant mitogenomes demonstrate a pronounced affinity for assimilating foreign DNA (Sprinzl and Vassilenko, 2005). Horizontal gene transfer (HGT) between the plant mitogenome and the nuclear genome is not only common but also a critical evolutionary driver (Christensen, 2013). For instance, split and partial gene transfer events involving the ribosomal protein gene *rpl2* have been identified in plant mitogenomes, providing direct evidence for the ease with which foreign DNA can be integrated into these genomes (Adams et al., 2001). Normally, the sequence exchange between plastomes and mitogenomes in plants has been perceived as predominantly one-directional, with the plastomes being the major donor. The chloroplast genome is highly conserved in higher plants, and few sequences from mitochondria have been found to be transferred into the plastid genome. However, research has indicated exceptions like *Asclepias syriaca* (Straub et al., 2013) and *Daucus carota* (Iorizzo et al., 2012) where mitochondrial sequences found their way into plastomes. Our study has unearthed sequences in the *P. crassipes* mitogenome that originated from its chloroplast genome (**Supplementary Table S8**; **Figure 4**). Among these, MTPT5, MTPT6, MTPT8, and MTPT9 are longer than 1,000 bp, with MTPT5 being the longest at 8,767 bp. These long MTPTs carry (partial) plastid PCGs to the mitogenome (Joyce and Gray, 1989; Oda et al., 1992; Chaw et al., 2008). Although these partial PCGs typically evolve into nonfunctional pseudogenes, they augment the diversity of mitochondrial DNA sources. A BLASTn analysis allowed us to pinpoint 7 tRNA genes in the *P. crassipes* mitogenome that migrated from the plastid to the mitochondria. These are two copies of *trnN-GUU*, *trnH-GUG*, *trnR-ACG*, *trnA-UGC*, *trnL-CAA*, and *trnW-CCA*. Over time, these transfer events have led to the acquisition of functional tRNAs that are conserved across angiosperms. Among these transferred tRNA genes, *trnW-CCA* is frequently observed in the mitogenomes of other angiosperms (Shan et al., 2023; Zhu et al., 2023; Jiang et al., 2023a).

Plant mitochondrial RNA editing represents a fascinating biological process in which specific nucleotides within the mitochondrial RNA sequence are transformed due to the actions of mitochondrial RNA editing enzymes (Wang et al., 2019; Liu et al., 2020; Wang et al., 2023a, b). These specialized enzymes are a distinct class of deaminases responsible for the C to U or U to C conversions within the RNA sequence (Gerke et al., 2020). In the realm of plants, RNA editing significantly influences cytoplasmic inheritance-related traits. More critically, it plays an indispensable role in mitochondrial gene expression and functionality (Sosso et al., 2012). Many of these RNA editing occurrences create new start and stop codons. These novel codons often code for proteins that display a higher degree of conservation and similarity to proteins in other species, optimizing gene expression within the mitochondria (Edera et al., 2018). In our research on *P. crassipes* mitogenome, we found a total of 765 RNA editing events. Interestingly, all these RNA editing sites were found at either the first or second positions, mirroring patterns seen in other plant species (Grewe et al., 2014; Kovar et al., 2018; Bi et al., 2020; Yang et al., 2021). The RNA editing frequency sequenced by Sanger sequencing seems to be lower than the expected frequency identified by lncRNA-seq data. As shown in **Supplementary Figure S4**, cytosine seems to be more dominant than thymine (uracil) at the expected RNA editing sites, but there is an obvious hybrid peak in the peak map of Sanger sequencing, which we speculate may be due to DNA contamination. In summary, the results here show significant RNA editing in partially sequenced mRNA, confirming the results in lncRNA-seq.

## Conclusion

In our study, we have accomplished the successful assembly of the mitogenome of *P. crassipes*, revealing a complex circular genome structure. We conducted thorough analyses to explore its gene content, repetitive elements, codon usage, MTPTs, and RNA editing sites, along with making phylogenetic inferences. To the best of our knowledge, this represents the first comprehensive description of a complete mitogenome within *P. crassipes*. Our findings provided valuable insights into the evolutionary history of mitogenomes.

## Data availability statement

The datasets presented in this study can be found in online repositories. The names of the repository/repositories and accession number(s) can be found in the article/**Supplementary Material**.

## Ethics statement

We collected fresh leaf materials of *E. crassipe* for this study. The study, including plant samples, complies with relevant institutional, national, and international guidelines and legislation. No specific permits were required for plant collection.

## Author contributions

ZH: Formal analysis, Funding acquisition, Methodology, Resources, Software, Validation, Visualization, Writing – original draft. XJ: Formal analysis, Funding acquisition, Resources, Writing – original draft. LP: Formal analysis, Methodology, Resources, Software, Writing – original draft. JG: Methodology, Software, Writing – original draft. YC: Formal analysis, Methodology, Software, Writing – original draft. JL: Funding acquisition, Supervision, Writing – review & editing. BL: Supervision, Validation, Writing – review & editing. AG: Supervision, Writing – review & editing. LL: Supervision, Writing – review & editing. RJ: Funding acquisition, Supervision, Writing – review & editing.

## Supplementary material

Supplementary File1 The aligned sequences of two *P. crassipes* mitogenomes.
