# Supplementary material for: The complete mitochondrial genome of Pontederia crassipes: using HiFi reads to investigate genome recombination and gene transfer from chloroplast genome
Source: Front Plant Sci. 2024 Jun 28;15:1407309. doi: 10.3389/fpls.2024.1407309 (PMC11240117; doi:10.3389/fpls.2024.1407309)

Supplementary Figures

Figure S1. The two potential configurations of *P. crassipes* mitogenome. **A**. shows a master circle of the complete *P. crassipes* mitogenome, and **B** shows an alternative configuration, including a minor circular contigs and a linear contigs.

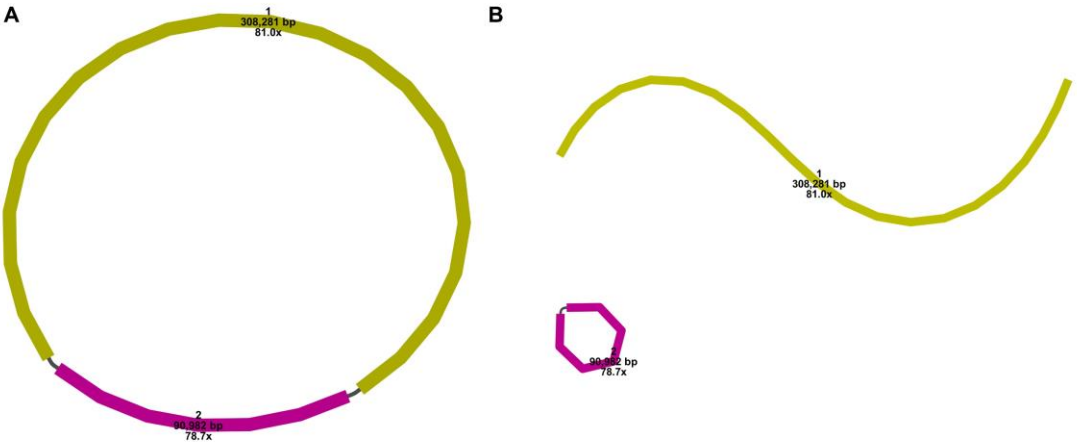

Figure S2. The Sanger sequencing results of the three paths in the mitogenome of *P. crassipes*. **A-C** represent the Sanger sequencing results compare to genomic DNA. The sequencing results are expected and verify the reality of these three paths, including c2-c1, c1-c2, and c2-c2.

A

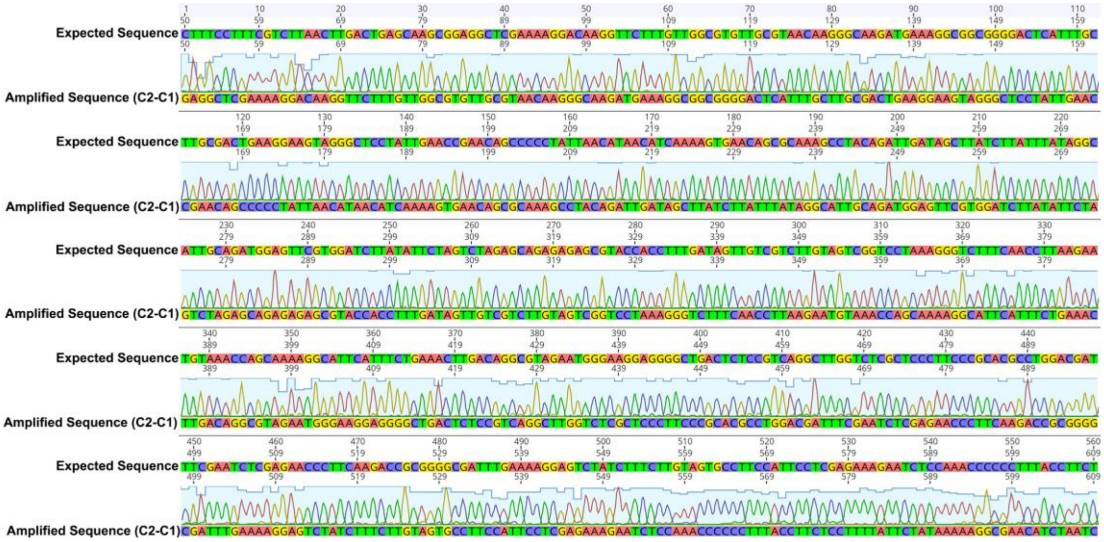

B

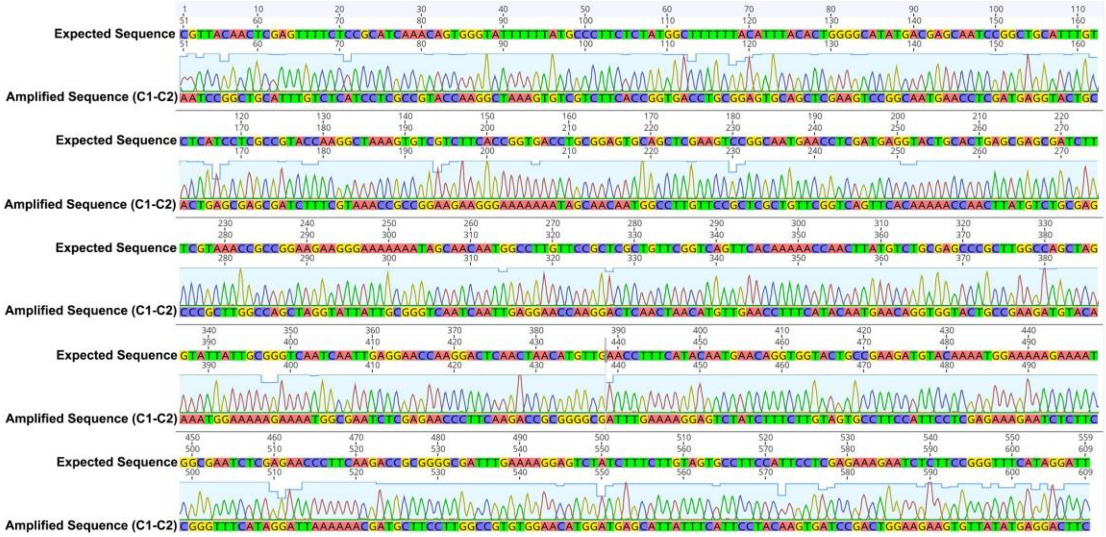

C

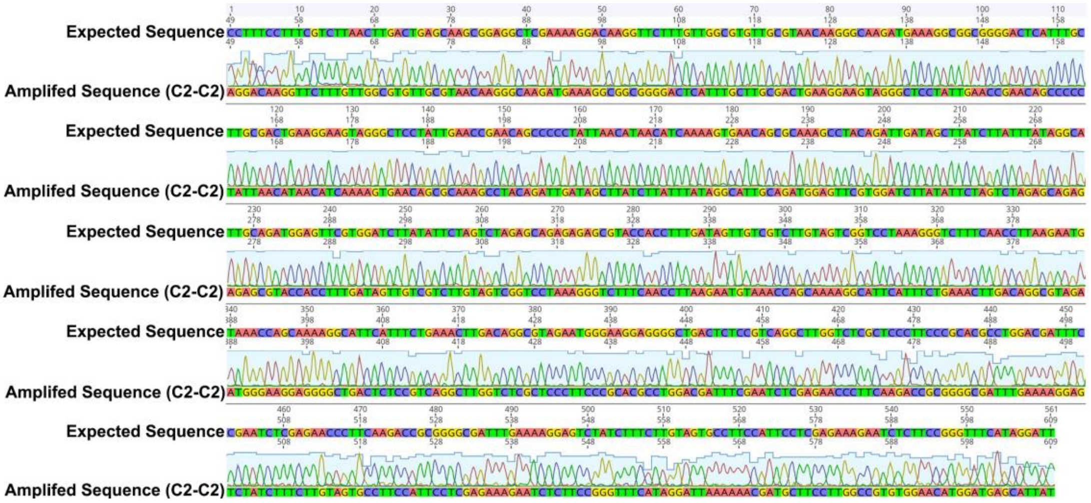
Expected Sequence
Amplifed Sequence (C2-C2)

Figure S3. Dot-plot comparison of OR680719.1/NC_084342.1 and PP112345.1. The horizontal and vertical coordinates respectively represent the genomic positions (bp). The red solid line indicates that the sequences have the same orientation, while the blue indicates that the orientations are opposite. Our results showed that the two mitogenomes are highly consistent.

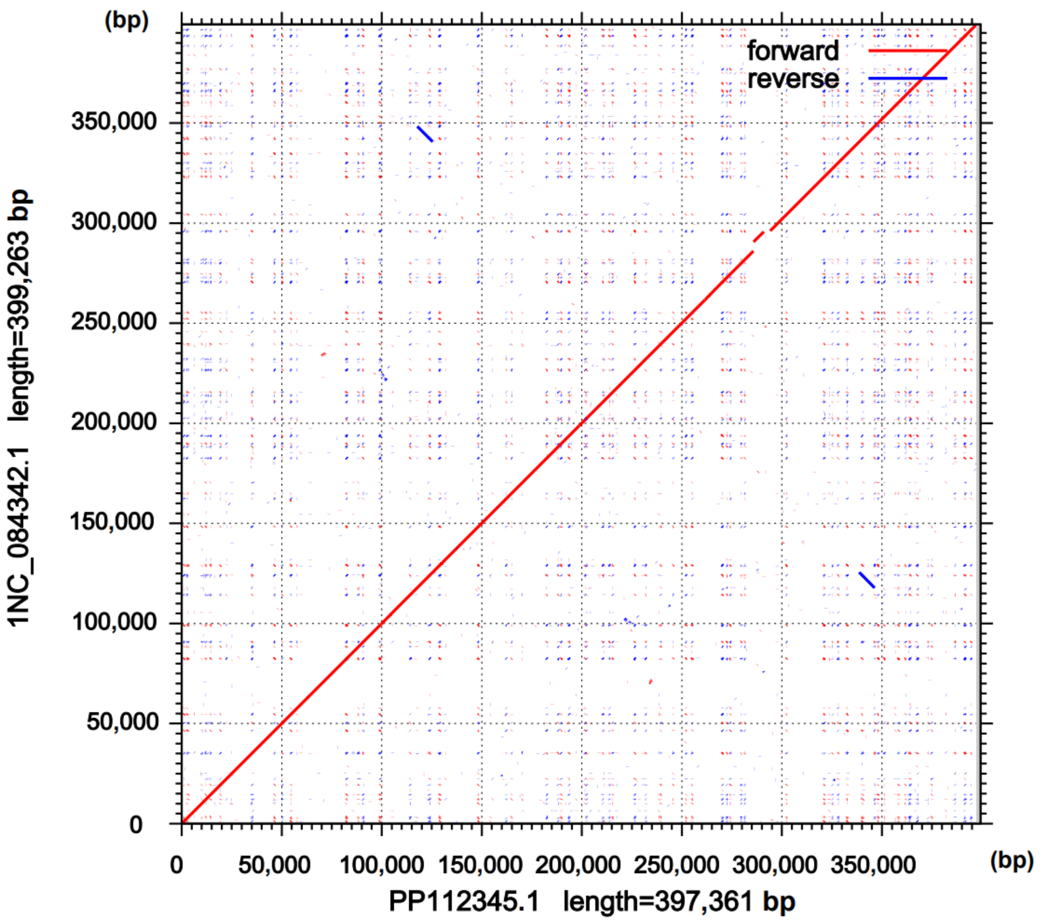

Figure S4. The codon usage and RSCU value of *P. crassipes* PCGs. Codon families are shown on the x-axis. RSCU values are the number of times a particular codon is observed relative to the number of times that codon would be expected for uniform synonymous codon usage.

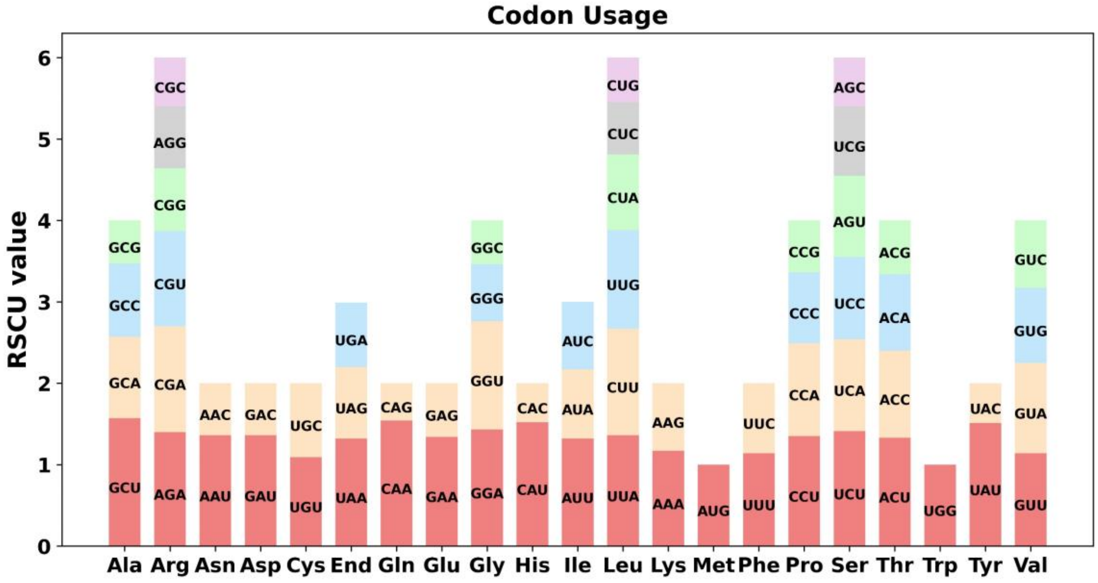

Figure S5. The PCR and Sanger sequencing results of random RNA editing site validation. **A.** show the results of PCR experiments on cDNA and gDNA of four genes. The experimental results are as expected. gDNA amplification length of *rpl10-2* gene is longer because of the presence of intron in the gene. **B.** shows the results of Sanger sequencing experiments on cDNA and gDNA of four genes, and it shows that RNA editing is not universally present in all cases.

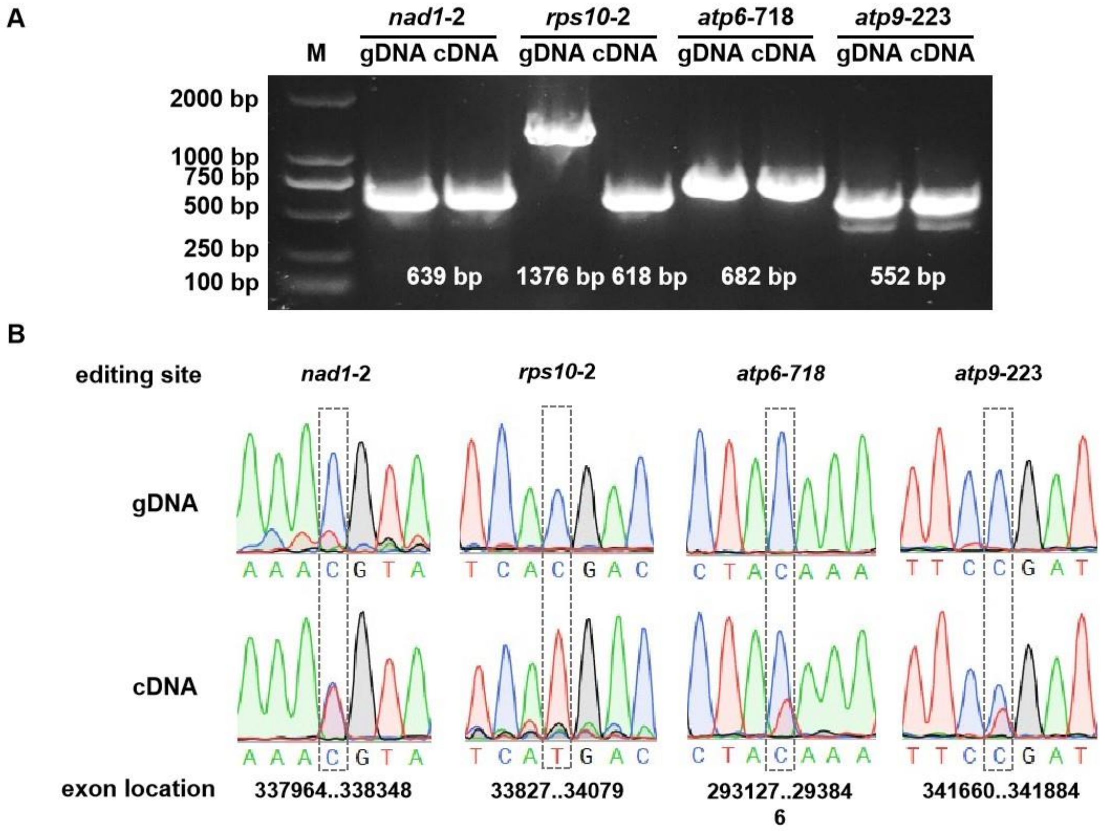

Supplement: Supplementary file 2 [file Image_1.pdf]
